# Ionic Liquids Catalysis for Carbon Dioxide Conversion With Nucleophiles

**DOI:** 10.3389/fchem.2018.00462

**Published:** 2018-10-08

**Authors:** Shu-Mei Xia, Kai-Hong Chen, Hong-Chen Fu, Liang-Nian He

**Affiliations:** ^1^State Key Laboratory and Institute of Elemento-Organic Chemistry, College of Chemistry, Nankai University, Tianjin, China; ^2^Collaborative Innovation Center of Chemical Science and Engineering, Nankai University, Tianjin, China

**Keywords:** CO_2_ conversion, carboxylative cyclization, catalysis, ionic liquids, green chemistry

## Abstract

Carbon dioxide, as a promising C_1_ synthon, has attracted great interest in organic synthesis. Due to the thermodynamic stability and kinetic inertness of CO_2_, developing efficient strategies for CO_2_ activation and subsequent conversion is very crucial. In this context, Ionic liquids (ILs) show great potential for capturing and activating CO_2_ owing to their unique structures and properties, making them become ideal alternatives to volatile organic solvents and/or catalysts for CO_2_ transformation. This minireview aims at summarizing ILs-promoted reactions of CO_2_ with *N*-nucleophiles (primary amines)/*O*-nucleophiles (primary alcohols, water). Two catalytic systems i.e., metal/ILs binary systems such as Cu/ILs systems and Ag/ILs systems as well as single ILs systems including anion-functionalized ILs and bifunctionalized ILs have been developed for CO_2_ catalytic conversion, for instance, carboxylative cyclization of nucleophiles e.g., propargylic alcohols, amines, 2-aminobenzonitriles and *o*-aminobenzenethiol, and formylation of amines or 2-aminothiophenols with hydrosilanes to afford various value-added chemicals e.g., cyclic carbamates, unsymmetrical organic carbonates, α-hydroxyl ketones, and benzimidazolones. In a word, IL could provide a powerful tool for efficient CO_2_ utilization.

## Introduction

CCS strategy, carbon capture and storage/sequestration, has been proposed as a most potential invention to reduce or mitigate CO_2_ emissions, including the capture of waste CO_2_, the transportation and deposition of CO_2_ in a safe place. Nevertheless, high cost and energy consumption of CCS process are the main obstacles. Carbon dioxide, as an abundant and non-poisonous C_1_ resource, has shown significant potential for constructing new C–C, C–O, and C–N bond in chemical synthesis (Shi et al., 2003; Zhang et al., 2008; He et al., 2009, 2010; Aresta et al., 2014; Liu et al., 2015, 2016a, 2017a,b; Song et al., 2017). However, the inherent thermodynamic stability and kinetic limitation of CO_2_ become the main barriers in transforming CO_2_ into high value-added chemicals, fuels, and materials. Therefore, developing efficient strategies for CO_2_ activation and conversion from environmental protection and economic perspectives is crucial. Carbon capture and utilization (CCU) strategy have been proposed by He group, which could be an ideal alternative to address the energy consumption problem in CCS (Yang et al., 2011a,b, 2012).

Ionic liquids (ILs) have attracted widespread attention as promising alternatives to solvents and catalysts on account of their unique properties such as the low melting point, unlimited tunability, negligible vapor pressure, and high stability (Zhang et al., 2006). As a novel green medium, ILs have been identified the outstanding performance in the absorption and conversion of CO_2_ under mild conditions through tunning the structures of cations and anions (Jutz et al., 2011; Yang et al., 2011b; Liu et al., 2016b). ILs-promoted CCU processes have attracted numerous attentions owning to ILs' unique properties. In most cases, ILs can be used as solvent, dehydrate, or catalyst, and these roles are similar in CCU processes. Thus, we hope to be able to shed light on all ILs-promoted CCU processes by using these limited but systematic examples.

In this minireview, we aim at summarizing ILs-promoted reactions of CO_2_ with some nucleophiles. We would like to divide this review into two parts that are metal/ILs binary systems such as Cu/ILs systems and Ag/ILs systems as well as single ILs systems including conventional ILs, anion-functionalized ILs, and bifunctionalized ILs. Selected carboxylative cyclization of nucleophiles, e.g., propargylic alcohols, amines, 2-aminobenzonitriles and *o*-aminobenzenethiol, and formylation of amines or 2-aminothiophenols with hydrosilanes, to afford various value-added chemicals are taken into consideration.

## Metal/ionic liquid binary catalytic system

**Scheme 1:**
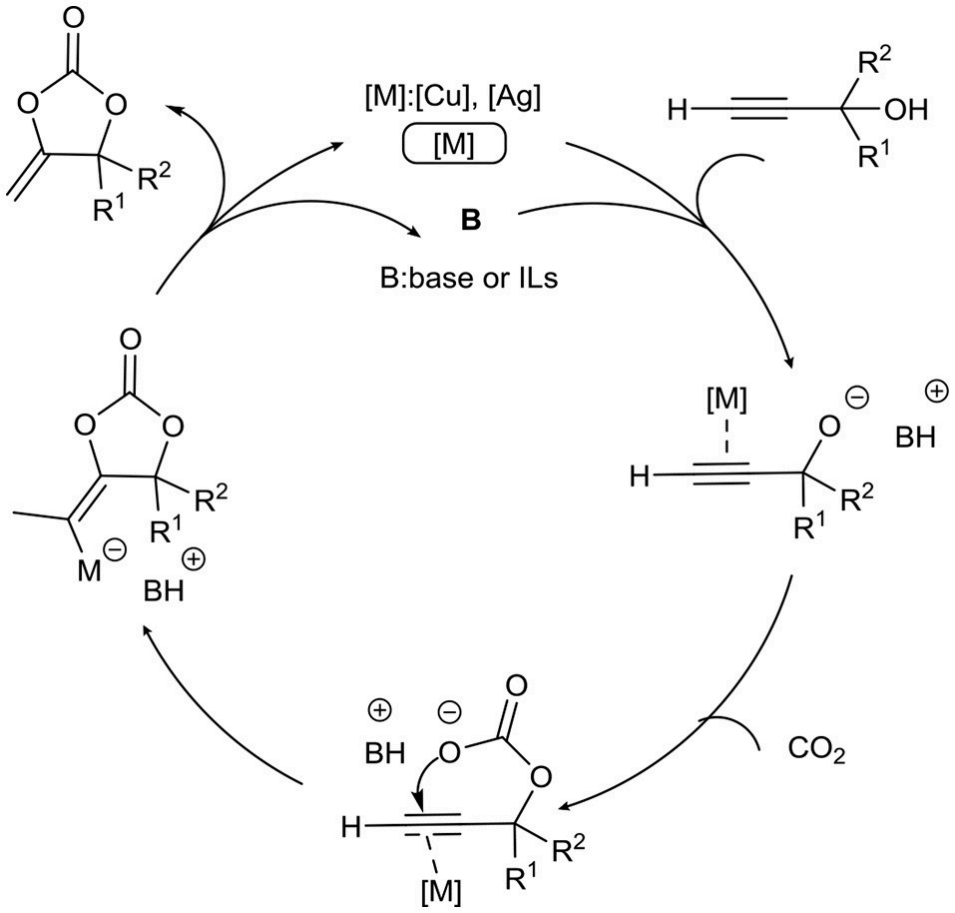
Plausible reaction mechanism of propargylic alcohols and CO_2_.

Metal (Cu, Ag)-ILs system is an important part of the CO_2_ capture and utilization. Since its ability to activate carbon-carbon triple bond, Cu/Ag has been used in various cyclization reactions of CO_2_ as shown in Scheme 1.

### Cu/ionic liquid catalysis

The first example of the synthesis of α-methylene cyclic carbonates from CO_2_ and propargylic alcohols in ILs is reported by Deng and co-workers (Gu et al., 2004; Figure S1). By screening of commercially available transition metal salts and ILs, [BMIm][PhSO_3_]/CuCl system exhibits the best performance in this reaction with a yield of 97%. Thanks to the reusability of ILs, CuCl can be reused 3 times without losing activity after immobilized in [BMIm][PhSO_3_].

A similar system, [BMIm]BF_4_/CuCl, is then used by the same group in a similar reaction (Figure S1; Gu et al., 2005). IL is used as the “green” reaction media and believed as the promoter for this three-component reaction of propargylic alcohols, primary amines and CO_2_.

The synthesis of α-methylene oxazolidinones only from propargylic amines and CO_2_ is reported by our group, in which bifunctional Cu(II)-polyoxometalate-based ILs is used as catalyst (Figure S2; Wang et al., 2016a). According to the experimental results and NMR studies, this IL is found to be able to activate propargylic amine and CO_2_ at the same time (Figure S3). And both terminal and internal propargylic amines successfully deliver the corresponding 2-oxazolidinones in excellent yields.

### Ag/ionic liquids catalysis

Compared with copper, silver displays better reactivity in activation of carbon-carbon triple bond of propargylic amine/ alcohol. Therefore, Ag-IL catalyst systems have also been widely applied for catalyzing cyclization propargylic amine/alcohols with CO_2_.

In 2015, He et al. successfully developed a dual-component catalytic system comprising AgOAc and [(*n*-C_7_H_15_)_4_N][Br], which could effectively catalyze CO_2_ fixation with propargylic alcohols/amines to produce various cyclic carbonates/oxazolidinones in the absence of solvent, ligand or organic base (Figure S4; Song and He, 2016). This elegant system can achieve the TON of 6024. Through experimental results and DFT calculations, the cation with a longer alkyl chain can enhance the nucleophilicity of anion resulting in improving its activity.

Besides of cations, tuning structures of anions in ILs is another way that can control the activity of ILs. Recently, Wang group has developed an efficient AgOAc/ [P_66614_][DEIm] (trihexyltetradecylphosphonium dimethyl 4,5-imidazoledicarboxylate) system for the synthesis of cyclic carbonates from propargylic alcohols with CO_2_ under mild conditions (Figure S5; Chen et al., 2016a). The basicity of the IL is found to play a dramatic role: only when IL with moderate basicity, can it have excellent activity. According to DFT calculation and NMR spectroscopic analyzing, weaker basicity shows poor activity but stronger basicity will reduce the yield because of the polymerization of propargylic alcohols.

Similarly, AgI/[OAc] is disclosed as a robust catalyst system in the same reaction (Figure S5; Yuan et al., 2017). Only 1% of the catalyst is needed to obtain excellent yield of cyclic carbonates. The high concentration of [OAc] in this system is favorable for the activation of hydroxyl in the substrate and CO_2_, which is also identified by Steckel (Steckel, 2012). Another Ag binary catalyst system, AgCl/[OAc], is reported by Han group in the reaction of CO_2_, propargylic alcohols, and primary alcohols (Figure S6; Hu et al., 2017). AgCl/[BMIm][OAc] serves as both catalyst and solvent, and can be easily reused at least five times without reducing notably catalytic activity. The activation of alcoholic hydroxyl by [OAc] and the activation of triple bond by Ag salt are supposed as important steps in this reaction.

## Ionic liquid catalysis for CO_2_ conversion

### Traditional ionic liquids

It has been a long time since ILs have been discovered the good solubility for CO_2_ (Blanchard et al., 1999; Bates et al., 2002; Jessop et al., 2005). And this property can also make IL a nice catalyst for CO_2_ conversion (Ma et al., 2017; Wang et al., 2018). Recently, two similar nonfunctional IL dual systems (CsOH or Co(acac)_3_/[BMMIm][Cl]) have been reported by Deng et al. in the synthesis of symmetric urea derivatives from CO_2_ and amines (Figure S7; Shi et al., 2003; Li et al., 2010). In the CsOH/[BMMIm][Cl] system, symmetric urea derivatives can be obtained in high yields, but no product is produced when without IL, indicating that IL is indispensable for this reaction. However, as a strong base, CsOH suffers from many weakness, such as corrosion, deactivation, and even destructive action to [BMMIm][Cl] under high temperature. In order to overcome these disadvantages, Co(acac)_3_/[BMIm][Cl] system is developed for this reaction. In this catalyst system, [BMIm][Cl] is believed as a physical dehydrant, importantly, it can be reused while keeping high activity.

IL, such as [BMIm][Br], is also believed as an efficient dehydrant in the synthesis of cyclic urethanes from 2-aminoethanol and CO_2_ (Figure S8; Fujita et al., 2006). K_2_CO_3_ is the catalyst in this reaction, while [BMIm][Br] acts as a recyclable dehydrant and can activate the carbonyl group. In another work to obtain oxazolidinone, Deng et al. developes an ILs-catalyzed efficient three-component reaction of propargylic alcohols, amines, and CO_2_ (Zhang et al., 2005). Among all of the solvents they use, [DMIm][BF_4_] exhibits the best performance, while traditional solvents like DMSO and toluene have no activity (Figure S9). Therefore, it is indicated that IL has a strong impact on this reaction. After recycling three times, high catalytic activity can be maintained for the [DMIm][BF_4_].

Imidazole IL ([BMIm][Cl]) is also applied by Liu group in the reductive functionalization of CO_2_ with amine**s** to afford formamide (Hao et al., 2015). The experimental results imply that both anions and cations of ILs play important roles in their activity. [BMIm][Cl] can activate not only the Si–H bond of phenylsilane to react with CO_2_, but the amine through the hydrogen bond. In addition, [BMIm][Cl] can be reused for five times with high activity (Figure S10).

### Anion-functionalized ionic liquids

**Scheme 2:**
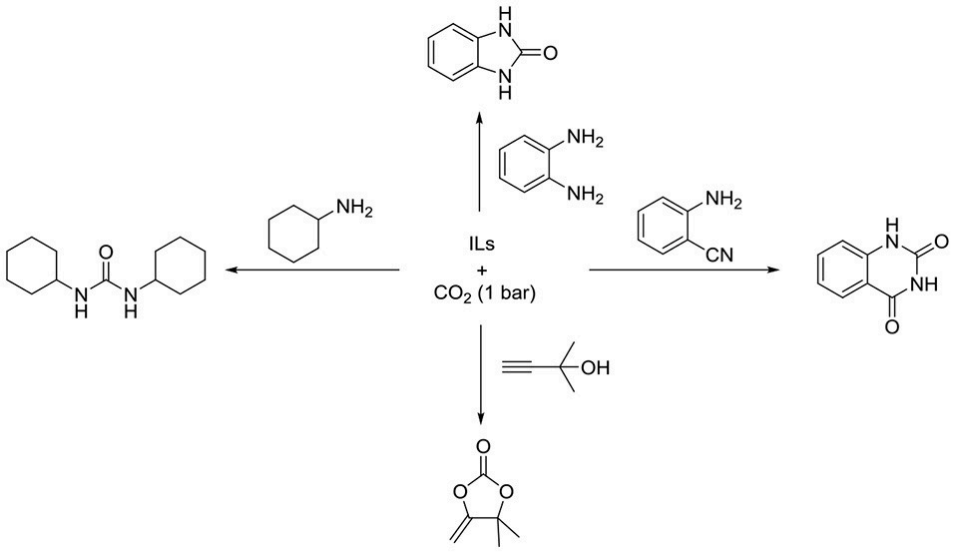
CO_2_ conversions catalyzed by IL_S_.

Although many CCU processes can be promoted by traditional ILs, most of them suffer from low ability for CO_2_ activation, in which high pressure (>1 MPa) or additional metal catalysts will be needed. Recently, anion-functioanlized ILs are designed for CO_2_ absorption and some of their CO_2_ absorption capacities are even 100 times higher than traditional organic solvent (Gurkan et al., 2010; Wang et al., 2011b; Yang et al., 2011a,b; Lei et al., 2014; Cui et al., 2016; Song et al., 2017). In addition, compared with traditional ILs, anion-functioanlized ILs also exhibit higher catalytic activities even without metal catalyst (Scheme 2).

In 2009, Patil et al. developed an alternative method that can obtain quinazoline-2,4(1H,3H)-diones easily from CO_2_ and 2-aminobenzonitriles by using [BMIm][OH] as catalyst (Patil et al., 2009). Except for [BMIm][OH], many inorganic bases, Et_3_N or traditional ILs ([BMIm][BF_4_] and [BMIm][HSO_4_]) show weak or no activity (Figure S11). For the role of [BMIm][OH] in this process, they propose that [OH] will activate the 2-aminobenzonitrile to initiate the reaction, while [BMIm] can stabilize the intermediate.

To elucidate the mechanism of this reaction, Wu et al. conducts a systematic DFT calculation (Ren et al., 2011). In the beginning, the mechanism proposed by Patil et al. is calculated to be energy unfeasible. Inspired by NHCs–CO_2_ adducts, they think these adducts might be a potential catalyst. Although the overall barrier is lower than the mechanism suggested by Patil et al. it is also too high to be taken into consideration. Then, a new mechanism with a lower energy barrier (42.5 kcal/mol) is proposed that [OH]^−^ initiated the reaction and the NHC, which is generated from [BMIm][OH], is the real catalyst for this process (Figure S12).

After that, in order to make catalyst's recycling easier, SiO_2_ supported [HMIm][OH] is used by Bhanage group in the reaction of 2-aminobenzonitriles and CO_2_ (Nale et al., 2014; Figure S11). This supported IL can be separated easily and only a minor change is found through FT-IR spectrum and surface areas analysis after using 3 times. Under optimized conditions, various electron-rich and electron-deficient groups on 2-aminobenzonitriles can react to give moderate to good yield.

Actually, imidazolium ILs are not stable under strong basicity conditions (Wang et al., 2017). Thus, many anions with weak basicity, such as acetate and azoles, are used instead of hydroxide. Han et al. discovers [Bmim][OAc] can catalyze the reaction of CO_2_ and 2-aminobenzonitriles even under atmospheric pressure of CO_2_ (Figure S13; Lu et al., 2014). Through screening different ILs, the anion in ILs is found to play a more significant role than the cation in this transformation. On the other hand, traditional organic/inorganic bases or non-functional ILs have no activity in this reaction.

Recently, Liu and coworkers utilize [BMIm][OAc] as catalyst in the cyclization of 2-aminothiophenols with CO_2_ and hydrosilane (Gao et al., 2015). A variety of benzothiazoles are obtained in moderate to excellent yield in the presence of [BMIm][OAc] and CO_2_ (Figure S13). Moreover, benzimidazoles can be also obtained in excellent yield by the same IL. Through ^1^H NMR analysis, [BMIm][OAc] is found to be capable of activating CO_2_, substrates and hydrosilane at the same time.

[BMIm][OAc] is found to promote the cross-link of chitosan by Guazzelli's group [Figure S13; (Mezzetta et al., 2017)]. Two amino groups in different chitosan chains can react with CO_2_ to form urea in [BMIm][OAc]. However, no cross-linked chitosan is found when using N-methyl-2-pyrrolidone as solvent, showing the importance of ILs. Moreover, the resulting products have good stability under different conditions that would have potential utility in drug delivery.

Azole-functionalized ILs are another kind of versatile ILs that can be utilized in the absorption of different gases (Wang et al., 2011a,b; Chen et al., 2015, 2016c; Cui et al., 2016). Also in the formation of urea, Deng and co-workers investigate the performance of various ILs in the reaction of CO_2_ with 1,6-hexamethylenediamine to form polyurea (Wang et al., 2016b). Since the importance of basicity of ILs is realized, [P_4446_][ATriz] is designed for this polymerization, and exhibits the best activity among the ILs used in this work (Figure S14).

Previous to this work, by using azole-functionalized ILs as catalysis, the group of Liu developes an efficient method to easily obtain α-ahydroxy ketones by the hydration of propargylic alcohol (Zhao et al., 2015; Figure S15). Azole-functionalized ILs are disclosed can promote this reaction even under atmospheric pressure of CO_2_. Based on the experimental and NMR investigations, the anion in IL, [Im]^−^, is proposed to capture CO_2_ at the first and then attack the triple bond of the propargylic alcohol to start the reaction.

In this mechanism, α-alkylidene cyclic carbonate is one of intermediates. Recently, the same group discovers that this reaction can stop at α-alkylidene cyclic carbonate when in an anhydrous conditions, while [P_4444_][2-MIm] showes the highest activity (Zhao et al., 2016; Figure S15). More importantly, the similar reaction condition can be also utilized in other reactions, such as CO_2_ with 2-aminobenzonitriles, *o*-phenylenediamines or 2-aminothiophenol. Since the ability to capture CO_2_ from atmosphere, [P_4444_][2-MIm] is believed to absorb CO_2_, then the formed carbamate intermediate would further react with substrates.

Although various kinds of substrates are suitable for this system, a high temperature (353 K) is needed to get α-alkylidene cyclic carbonate in that work. To lower down the energy demanding, they then use a new IL, [Bu_4_P]_3_[2,4-OPym-5-Ac], as catalyst in the cyclization reaction of propargylic alcohols with CO_2_ at ambient conditions (Wu et al., 2017; Figure S15). In addition, cations in ILs also exhibit a significant effect on this transformation, only the quaternary ammonium with C_4_ chain can it have good activity.

Apart from these ILs, tungstate ILs are found to be also able to catalyze this reaction under mild conditions (Kimura et al., 2012b). Mizuno group calculates the natural bond orbital (NBO) charges of O on different tungstates at the first, and [WO_4_]^−^ is found to have more negative charges than others, suggesting that it should be the most basic among these tungstates. Then, in the reaction of 1,2-phenylenediamine with CO_2_ to give 2-benzimidazolone, [N_4444_]_2_[WO_4_] exhibits the best performance. Importantly, quinazoline-2,4(1H,3H)-diones, cyclic carbonates, and urea derivatives can be also obtained in good to excellent yield by using [N_4444_]_2_[WO_4_] as catalyst. Through ^1^H, ^13^C and ^183^W NMR spectra analysis, [WO_4_]^−^ is identified can activate not only substrates but CO_2_. After that, this dual-functional role of [WO_4_] is also verified by DFT calculation in the reaction of CO_2_ and 2-aminobenzonitriles (Kimura et al., 2012a).

### Bifunctionalized ionic liquids

As noted above, anion-functionalized ILs can promote the CO_2_ utilization because of their basicity. In many cases, however, cations in ILs are also helpful to lower down the energy barriers through forming hydrogen bond, and we call this kind of ILs as bifunctional ILs. Liu et al. have reported protic IL (PIL), [HDBU^+^][TFE], as catalyst that can simultaneously activate CO_2_ and aminobenzonitriles to synthesize of quinazoline-2,4(1H,3H)-diones (Zhao et al., 2014). In the mechanism analysis, the hydrogen bond between [HDBU] and substrates is found to facilitate the nucleophilic attack of substrates to CO_2_, which is activated by [TFE].

Using a similar IL, [DBUH][OAc], they disclose that *o*-phenylenediamines can react with CO_2_ to obtain benzimidazolones in mild condition (Yu et al., 2013). In this reaction, three DBU-ILs with different anion and [*n*-Bu-DBUH][OAc] are synthesized, then the activity is showed in the order: [DBUH][Cl] < [*n*-BuDBU][OAc] < [DBUH][Lac] < [DBUH][OAc]. NMR spectra demonstrate that [DBUH][OAc] acts as a bifunctional catalyst: the cation [HDBU] activated effectively CO_2_, while the nucleophilicity attack of *o*-phenylenediamine to CO_2_ is enhanced by hydrogen bond with [OAc] (Figure S16).

In 2014, Zheng et al. reported an IL-catalyzed route to fix CX_2_ (O, S) with 2-aminobenzonitriles for the synthesis of quinazoline-2,4(1H,3H)-diones and quinazoline-2,4(1H,3H)-dithiones (Zheng et al., 2014). The ILs can be formed via the mixture of DBU and ethanol under bubbling atmospheric pressure of CO_2_ or CS_2_ (Figure S17), which act as both catalyst and solvent in CX_2_ (O, S) conversion. As shown in previous, both of the cation and anion in this IL are significant in this reaction (Figure S18).

In 2015, Han and coworkers successfully developed a new method of synthesizing a series of 2-oxazolidinones from atmosphere CO_2_ and propargylic amines by utilizing [DBUH][MIm] as both catalyst and solvent under mild conditions [Figure S19; (Hu et al., 2015)]. Based on the DFT investigation, [MIm]^−^ can capture and activate CO_2_, at the same time, the H atom on [DBUH]^+^ can attack the triple bond, which promotes effectively the intramolecular cyclization step (Figure S20).

Subsequently, Wang and co-workers firstly realize ILs-catalyzed one-pot domino hydration of diyne alcohols to synthesize 3-(2H)-furanones (Figure S21), where H_2_O acts as both a substrate and solvent under atmospheric pressure CO_2_ (Chen et al., 2016b). This is the first time to predict the catalytic activity of ILs through quantum calculation, and [HDBU][BenIm] shows more effective on catalytic activity than other ILs. Based on NMR spectroscopic investigation and DFT calculation, both cations and anions in ILs are important to keep a moderate basicity so as to reach an excellent reactivity.

Apart from DBU-based ILs, He et al. report carboxylative cyclization of 2-aminobenzonitriles with ambient CO_2_ by employing [HTMG][Im], as highly efficient and recyclable catalyst (Lang et al., 2016). This system has a broad substrate tolerance, and [HTMG][Im] exhibits excellent reusability.

Recently, Wang et al. developed an efficient strategy for generation of quinazoline-2,4(1H,3H)-diones from CO_2_ through varying the cations to design hydroxyl functionalized ILs, in which the catalytic activity is affected by the basicity of cation and the hydrogen bond from cation shows great promotion for this reaction (Shi et al., 2018). The aprotic IL (2-hydroxyethyl)-trimethyl-ammonium imidazole, [Ch][Im], gives the best catalytic activity. In addition, the high yield of quinazoline 2,4-(1H,3H)-dione can be obtained under one-gram scale and flue gas simulation system using [Ch][Im] as catalyst.

## Conclusion and outlook

As a sustainable C_1_ source, CO_2_ capture and utilization is an attractive field in view of environmental protection. Lots of strategies have been developed for the utilization of CO_2_, and the products have potential utility in biology and pharmacy. However, there is still a long way for most of these reactions to meet the requirement of industry, because of high cost and low efficiency. ILs-promoted CCU processes have attracted numerous attentions owning to ILs' unique properties. In most cases, ILs can be used as solvent, dehydrate, or catalyst, and these roles are similar in CCU processes. Thus, we hope to be able to shed light on all ILs-promoted CCU processes by using these limited but systematic examples. Owing to the nature of ILs, CO_2_-philic functional groups, such as oxygen atoms, and amine groups, could be incorporated into the anion and/or cation in ILs. Functional ILs show great potential for the absorption and conversion of CO_2_, and have been used as solvent, dehydrate, or catalyst in CCU processes. The anion-functionalized ILs exhibit better catalytic effect than the functionalization of cation part. We believe that ILs-promoted CCU protocol provides high efficient conversion of CO_2_ to synthesize a series of oxazolidinones, ureas, etc. We believe this ILs-promoted CCU protocol will be widely applied in CO_2_ chemistry, especially chemical utilization of CO_2_ to produce added-value commodity chemicals in industry.

However, a few of interesting or important fields are still needed to explore. At first, the efficiency of these processes needs to be improved, either new efficient CO_2_ utilized reaction or highly efficient and low-cost catalyst needs to be discovered. On the other hand, decreasing the cost of these reactions, such as lowering the pressure and temperature or using renewable energy (light or electricity), is a tendency for these researches. In addition, structures of ILs are related to their properties, which exhibit dramatically influence on their activity. Therefore, the investigation of the way structures affects their activity should have a profound significance in this field. Only when we obtain these answers, can we know how to design an efficient IL for specific reaction without basing on experience and experimental trial and error.

## Author contributions

All authors contributed for the writing of the manuscript. L-NH designed this proposal and determined the contents. S-MX wrote the Abstract, Introduction, Ag/Ionic Liquids Catalysis, and Bifunctionalized Ionic Liquids. H-CF wrote the Cu/Ionic Liquid Catalysis and Traditional Ionic Liquids Parts. K-HC wrote Anion-Functionalized Ionic Liquids, Conclusion and Outlook. K-HC, L-NH, and S-MX revised the manuscript.

### Conflict of interest statement

The authors declare that the research was conducted in the absence of any commercial or financial relationships that could be construed as a potential conflict of interest.

## Abbreviations

[BMIm][PhSO_3_]: 1-butyl-3-methylimidazolium benzenesulfonate
[BMIm][BF_4_]: 1-*n*-butyl-3-methylimidazolium tetrafluoroborate
[(*n*-C_7_H_15_)_4_N][Br]: tetraheptylammonium bromide
[P_66614_][DEIm]: trihexyl(tetradecyl)phosphonium dimethyl 4,5-imidazoledicarboxylate
[Bmim][OAc]: 1-butyl-3-methylimidazolium acetate
[Bmim][Cl]: 1-*n*-butyl-3-methyl imidazolium chloride
[BMIm][Br]: 1-*n*-butyl-3-methyl imidazolium bromide
[DMIm][BF_4_]: 1-*n*-decyl-3-methylimidazolium tetrafluoroborate
[Bmim][OH]: 1-*n*-butyl-3-methylimidazolium hydroxide
[BMIm][HSO_4_]: 1-*n*-butyl-3-methylimidazolium hydrosulfate
[HMIm][OH]: 1-hexyl-3-methylimidazolium hydroxide
[P_4446_][ATriz]: hexyltributylphosphonium aminotriazole
[Bu_4_P]_3_[2,4-OPym-5-Ac]: tritetrabutylphosphonium 2-oxidopyrimidine-5-carboxylate
[N_4444_]_2_[WO_4_]: tetrabutylamine tungstate
DBU: 1,8-diazabicyclo[5.4.0]undec-7-ene
TFE: trifluoroethanol
[DBUH][TFE]: 1,8-diazabicyclo[5.4.0]undec-7-ene trifluoroethanol
[DBUH][OAc]: DBU acetate
[DBUH][Lac]: DBU lactate
[DBUH][Cl]: DBU chloride
[*n*-Bu-DBUH][OAc]: *n*-butyl DBU acetate
[DBUH][MIm]: DBU 2-methylimidazolide
[HDBU][BenIm]: DBU Benzimidazole
[HTMG][Im]: 1,1,3,3-tetramethylguanidinium imidazolide
[Ch][Im]: (2-hydroxyethyl)-trimethyl-ammonium imidazole.
