# Supplementary material for: Ionic Liquids Catalysis for Carbon Dioxide Conversion With Nucleophiles
Source: Front Chem. 2018 Oct 8;6:462. doi: 10.3389/fchem.2018.00462 (PMC6186839; doi:10.3389/fchem.2018.00462)
Supplement: Supplementary file 1 [file Data_Sheet_1.pdf]

Supplementary Material

Ionic liquids Catalysis for Carbon Dioxide Conversion with Nucleophiles

Shu-Mei Xia , Kai-Hong Chen , Hong-Chen Fu , Liang-Nian He [*]

*** Correspondence:** Liang-Nian He Email: heln@nankai.edu.cn; Tel.: +86-22-23503878.

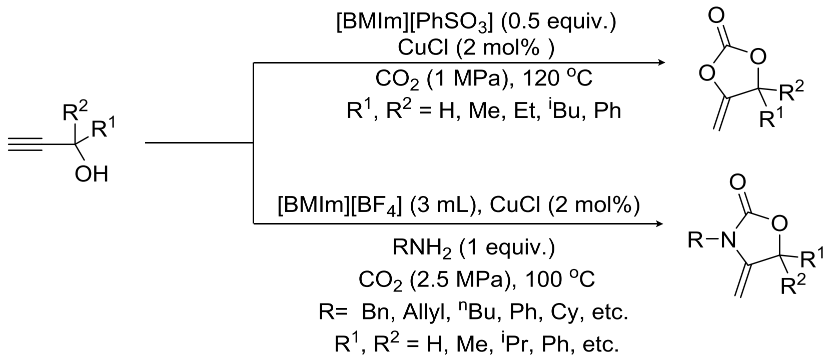

FIGURE S1 | Cu-catalyzed reaction of propargylic alcohols with CO_2.

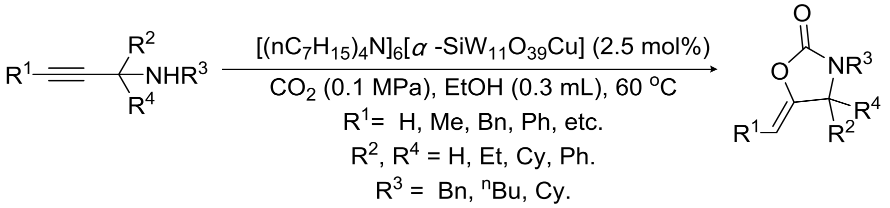

FIGURE S2 | Synthesis of oxazolidinones using Cu/ILs.

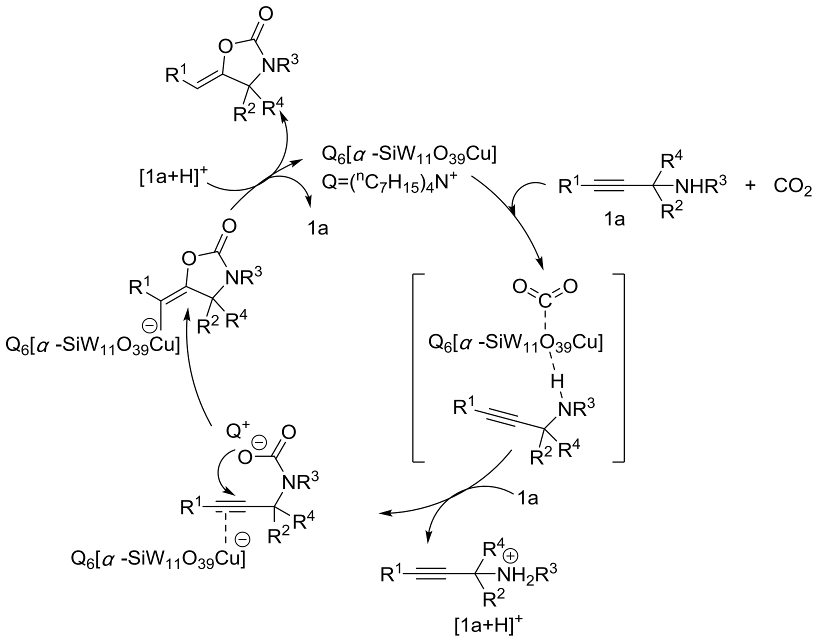

FIGURE S3 | Plausible mechanism by using Cu/ILs as catalyst.

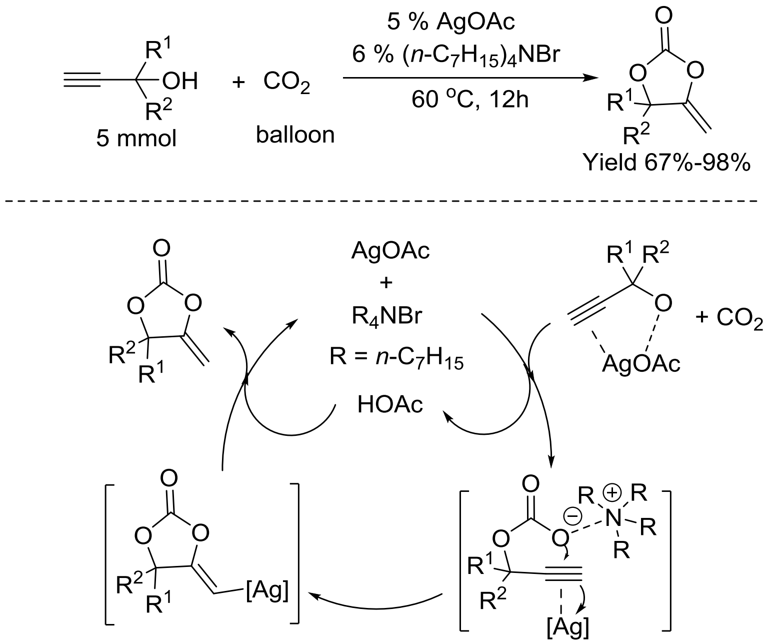

FIGURE S4 | Plausible reaction mechanism.

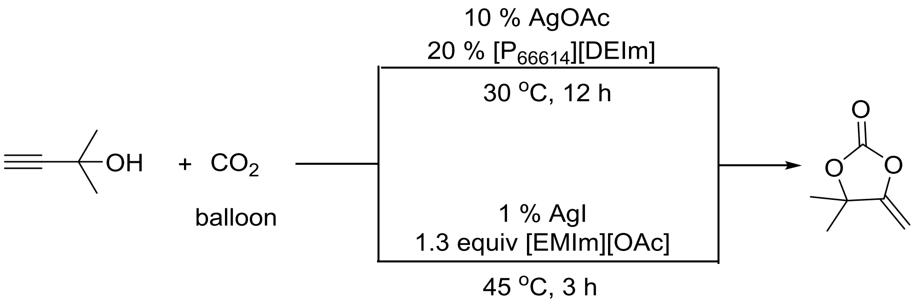

FIGURE S5 | Reaction of CO_2 and propargylic alcohols by using Ag/IL system.

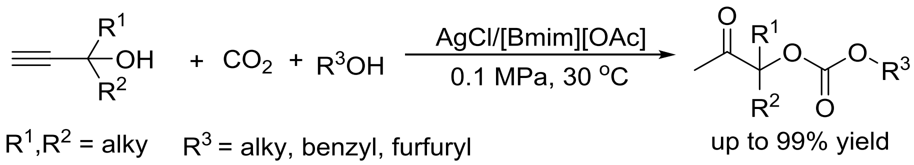

FIGURE S6 | Reaction of CO_2, propargylic alcohols, and primary alcohols to synthesize asymmetrical organic carbonates.

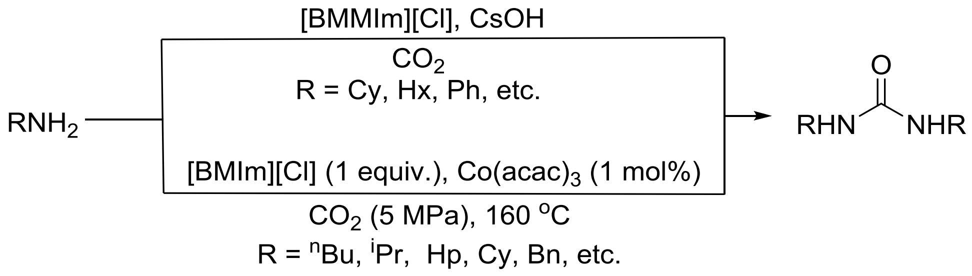

FIGURE S7 | Synthesis of urea in ILs.

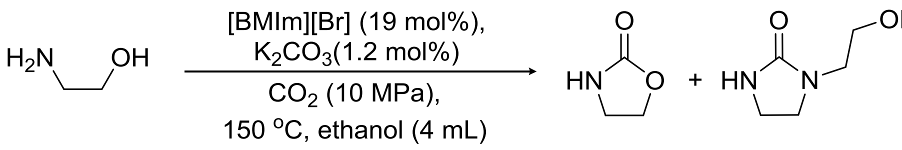

FIGURE S8 | Synthesis of oxazolidinones in ILs.

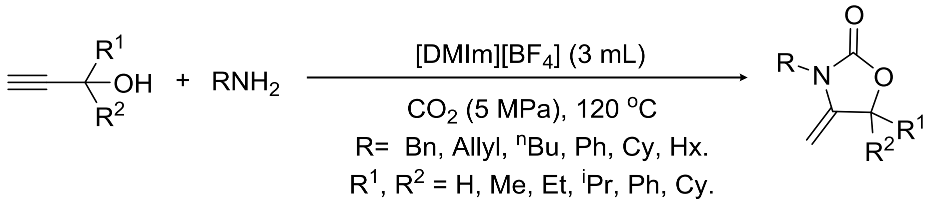

FIGURE S9 | ILs-catalyzed three-component reaction of propargylic alcohols, amines, and CO_2.

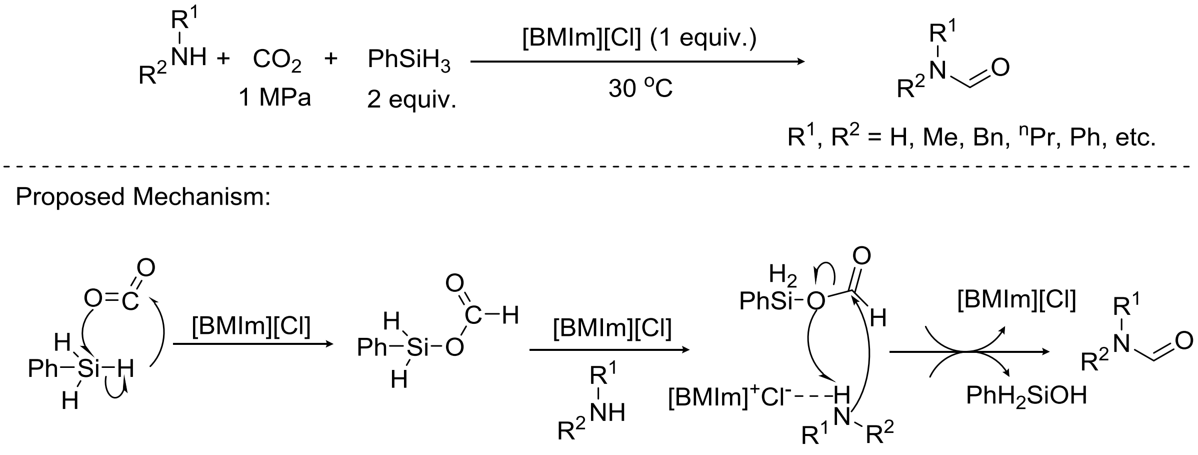

FIGURE S10 | Reductive functionalization of CO_2 with amines in ILs.

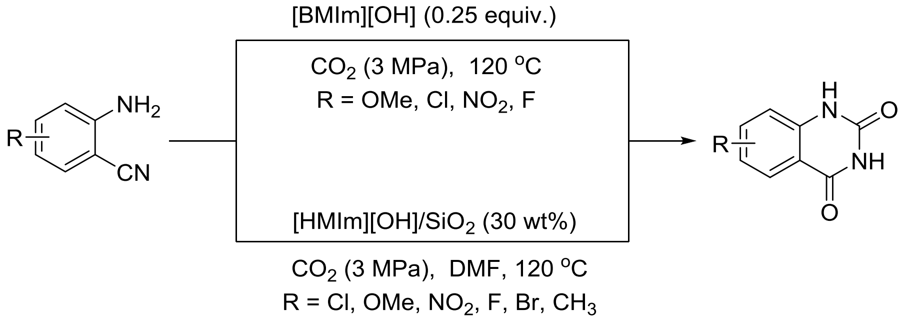

FIGURE S11 | ILs-catalyzed reaction of 2-aminobenzonitriles with CO_2.

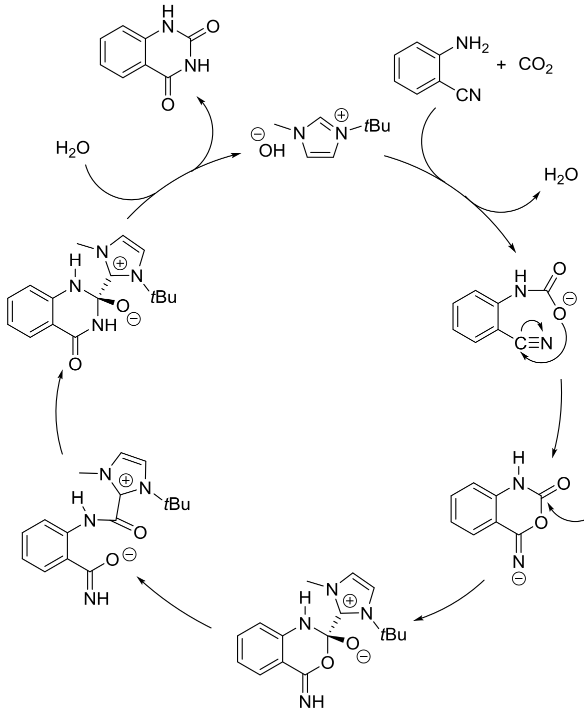

FIGURE S12 | The mechanism proposed by Wu group.

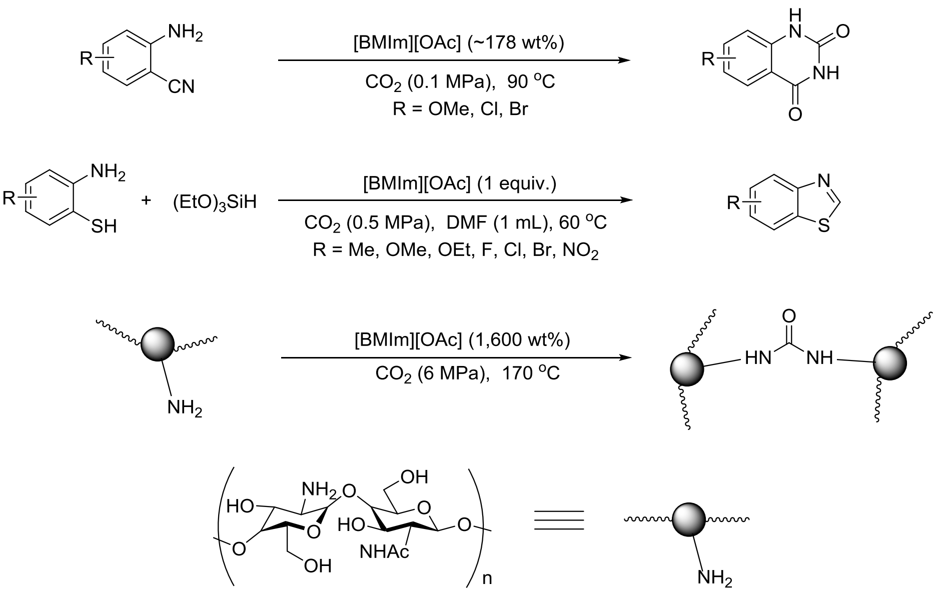

FIGURE S13 | Typical reactions catalyzed by [BMIm][OAc].

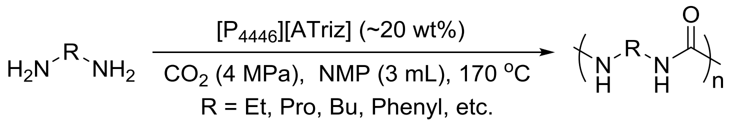

FIGURE S14 | Synthesis of polyurea using [P_{4446}][ATriz].

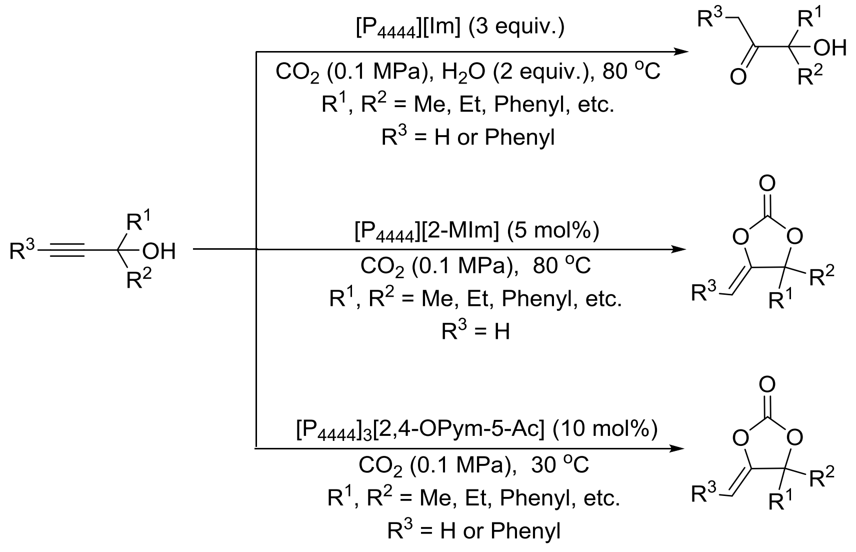

FIGURE S15 | Reaction of propargylic alcohol and CO_2 under different conditions.

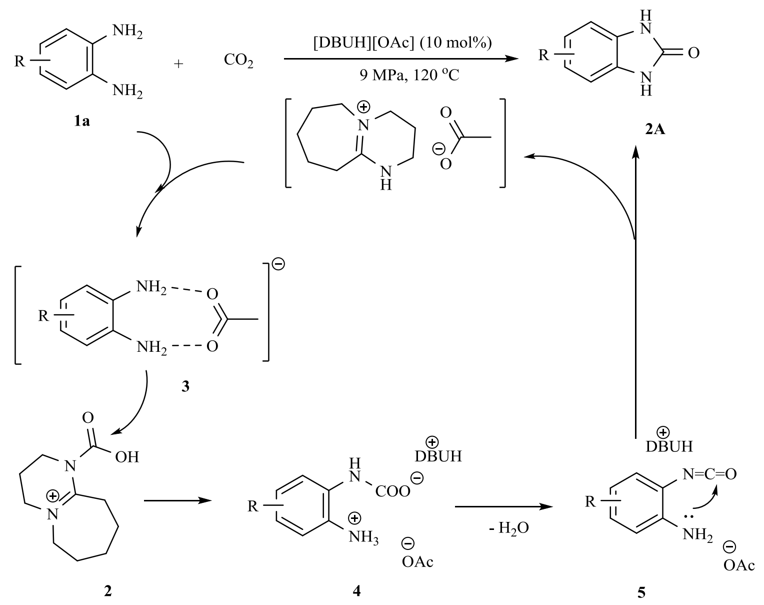

FIGURE S16 | Carbonylative cyclization of vicinal diamines with CO_2.

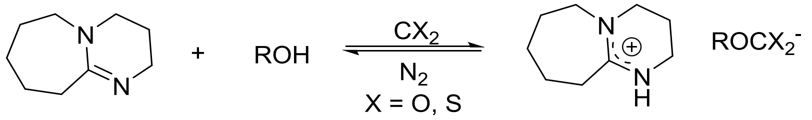

FIGURE S17 | The synthesis of two types of ILs by capturing CX_2 (O, S).

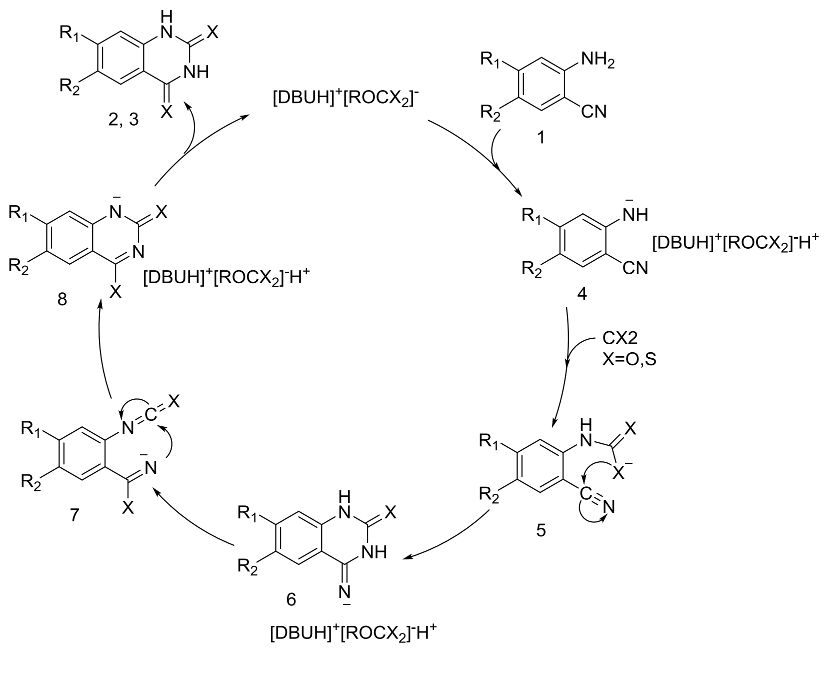

FIGURE S18 | A plausible mechanism for the capture of CX_2 (O, S) by synthesizing quinazoline derivatives in ReILs.

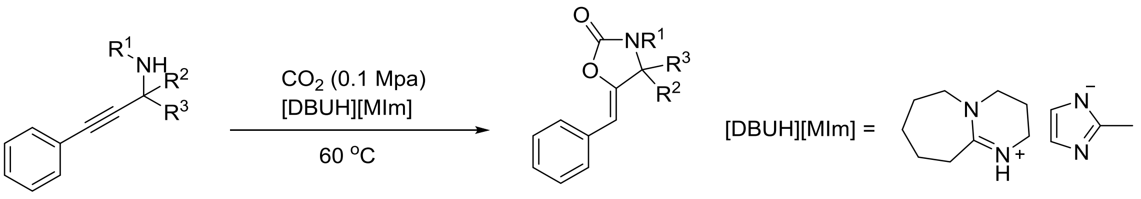

FIGURE S19 | Reaction of CO_2 with propargylic amine in ILs.

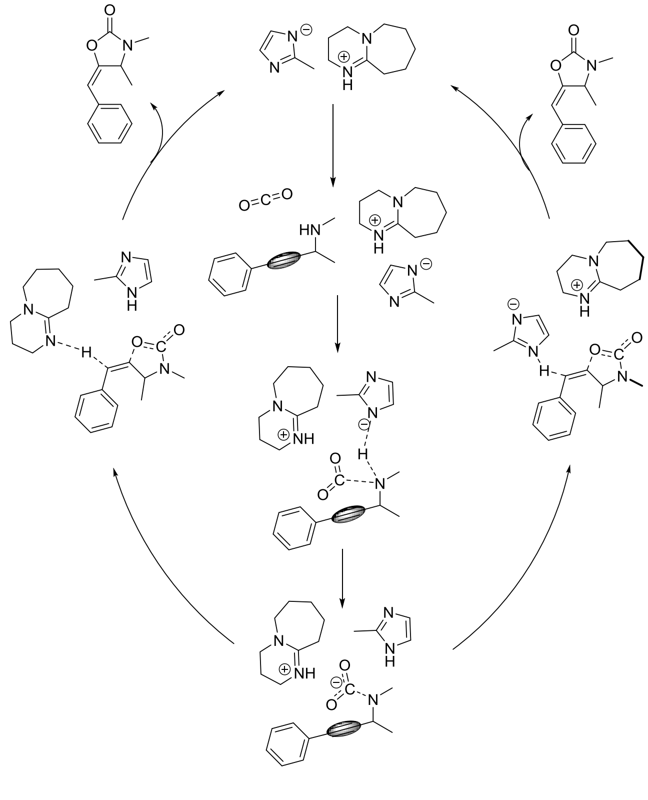

FIGURE S20 | The mechanism proposed by Han group.

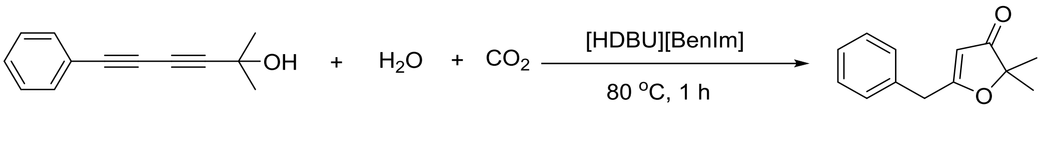

FIGURE S21 | Reaction of CO_2 with diyne alcohols in [HDBU][BenIm].
